# DDR1 Affects Metabolic Reprogramming in Breast Cancer Cells by Cross-Talking to the Insulin/IGF System

**DOI:** 10.3390/biom11070926

**Published:** 2021-06-22

**Authors:** Veronica Vella, Marika Giuliano, Maria Luisa Nicolosi, Maria Giovanna Majorana, Małgorzata Anna Marć, Maria Grazia Muoio, Andrea Morrione, Marcello Maggiolini, Rosamaria Lappano, Ernestina Marianna De Francesco, Antonino Belfiore

**Affiliations:** 1 Unit of Endocrinology, Department of Clinical and Experimental Medicine, University of Catania, Garibaldi-Nesima Hospital, 95122 Catania, Italy; veronica.vella@unict.it (V.V.); marika.giuliano@phd.unict.it (M.G.); nicolosi.ml@gmail.com (M.L.N.); mariagiovanna.majorana@gmail.com (M.G.M.); marcmalgorzata@gmail.com (M.A.M.); mariagrazia.muoio@unict.it (M.G.M.); ernestina.defrancesco@unict.it (E.M.D.F.); 2 Center for Biotechnology, Department of Biology, Sbarro Institute for Cancer Research and Molecular Medicine, College of Science and Technology, Temple University, Philadelphia, PA 19122, USA; Andrea.Morrione@temple.edu; 3 Department of Pharmacy, Health and Nutritional Sciences, University of Calabria, 87036 Rende, Italy; marcello.maggiolini@unical.it (M.M.); rosamaria.lappano@unical.it (R.L.)

**Keywords:** discoidin domain receptor 1 (DDR1), metabolic reprogramming, tumor metabolism, insulin, hyperinsulinemia, insulin receptor (IR), insulin receptor isoform A (IR-A), insulin-like growth factor receptor 1 (IGF1R), insulin growth factor 2 (IGF2), tumor matrix

## Abstract

The insulin receptor isoform A (IR-A), a dual receptor for insulin and IGF2, plays a role in breast cancer (BC) progression and metabolic reprogramming. Notably, discoidin domain receptor 1 (DDR1), a collagen receptor often dysregulated in cancer, is involved in a functional crosstalk and feed forward loop with both the IR-A and the insulin like growth factor receptor 1 (IGF1R). Here, we aimed at investigating whether DDR1 might affect BC cell metabolism by modulating the IGF1R and/or the IR. To this aim, we generated MCF7 BC cells engineered to stably overexpress either IGF2 (MCF7/IGF2) or the IR-A (MCF7/IR-A). In both cell models, we observed that DDR1 silencing induced a significant decrease of total ATP production, particularly affecting the rate of mitochondrial ATP production. We also observed the downregulation of key molecules implicated in both glycolysis and oxidative phosphorylation. These metabolic changes were not modulated by DDR1 binding to collagen and occurred in part in the absence of IR/IGF1R phosphorylation. DDR1 silencing was ineffective in MCF7 knocked out for DDR1. Taken together, these results indicate that DDR1, acting in part independently of IR/IGF1R stimulation, might work as a novel regulator of BC metabolism and should be considered as putative target for therapy in BC.

## 1. Introduction

Discoidin domain receptor 1 (DDR1) is a tyrosine-kinase receptor activated by fibrillar collagen type I but also collagens type II, III, IV, and V [1,2]. DDR1 plays a complex role in development and organogenesis as well as in inflammation and fibrosis [3,4,5]. Moreover, DDR1 is often dysregulated in cancer, and it has been implicated in various aspects of cancer progression, including cell proliferation and invasion, promotion of stem phenotype, metastasis, and modulation of chemotherapy response [6,7]. We have recently described a feed-forward loop between DDR1 and the insulin/insulin-like growth factor signaling (IIGFs) [8]. DDR1 was critical for IGF-IR endocytosis and trafficking into early endosomes, and DDR1 overexpression induced upregulation of both the IGF1 receptor (IGF1R) and the insulin receptor (IR) in normal and tumor cells. Notably, DDR1 preferentially modulated the expression of the oncofetal IR isoform IR-A by altering the balance of IR isoforms and favoring a high IR-A:IR-B ratio [9,10]. It also stimulated the secretion of IGF2, a shared ligand of IR-A and IGF1R. In turn, IIGF activation induced DDR1 upregulation and phosphorylation. Notably, DDR1 enhanced breast cancer cell proliferation, migration, and colony formation in response to insulin and IGFs [11,12,13,14]. Moreover, we demonstrated that DDR1 promotes thyroid cancer cell dedifferentiation and the acquisition of a stem-like phenotype by enhancing the IGF-2/IR-A autocrine signaling loop [9,10] and plays a critical role in promoting bladder cancer cell motility by linking the IGF1R and IR-A to the regulation of F-actin cytoskeleton dynamics [15]. Collectively, these data uncover novel, non-canonical DDR1 functions, which strongly support the pro-tumorigenic and pro-metastatic role of DDR1 [16,17,18].

Metabolic reprogramming is a hallmark of cancer and fundamental for cancer cells in order to achieve limitless cell proliferation and escape from apoptosis [19,20]. A common characteristic of this process is the preference of cancer cells for aerobic glycolysis, an inefficient mechanism of glucose metabolism that requires high glucose uptake and production of large amounts of lactate. This feature, known as the Warburg effect [21,22], allows increased synthesis of intermediates for sustaining anabolic pathways critical for cancer cell growth. In addition, cancer cells transform their mitochondria into synthesis machines supported by augmented glutaminolysis, lipid production, amino acid synthesis, and pentose phosphate pathways [23]. Whether DDR1 can affect metabolic reprogramming of cancer cells is currently unknown. Notably, we have recently demonstrated that IIGFs activation might contribute to the metabolic reprogramming of breast cancer (BC) cells [8]. In particular, human MCF7 BC cells, knocked out for IGF1R and stably overexpressing human oncofetal IR-A (MCF7^IGF1R-ve^/IR-A), acquired increased aerobic glycolysis associated with increased mitochondrial biogenesis and activity, which promoted high cell proliferation and invasion when exposed to either insulin or IGF2 [8]. MCF7 cells constitutively overexpressing human IGF2 (MCF7/IGF2) showed similar metabolic changes. Based on these findings, we hypothesized that DDR1, as a critical regulator of IIGFs, might also play a role in the metabolic reprogramming of BC cells. 

## 2. Materials and Methods 

### 2.1. Materials

Bovine serum albumin (BSA) was obtained from Sigma-Aldrich (Saint Louis, MO, USA); non-fat dry milk from Santa Cruz Biotechnology (Santa Cruz, CA, USA); Opti-MEM, fetal calf serum (FCS), TRIzol Reagent, ThermoScript RT kit, SYBR Green MasterMix, and lipofectamine RNAiMax from Life Technologies, Inc. Laboratories (Paisley, UK); and nitrocellulose membranes and HRP-conjugated secondary antibodies from Amersham Biosciences (Little Chalfont, UK). MitoTracker^®^ Orange CMTMRos was from ThermoFisher (Waltham, MA, USA). IGF2 ELISA kit E30 was from Mediagnost, Reutlingen, Germany (minimum detectable concentration: 0.02 ng/mL, inter- and intra-assay variation coefficients are less than 7.2% and 6.6% respectively). 

### 2.2. Cell Cultures 

The human cancer cell line MCF7 was purchased from the American Cell Type Culture Collection (ATCC) and cultured according to the manufacturer’s instructions. Cells were grown in complete MEM (Sigma, St. Louis, MO, USA) supplemented with 10% fetal bovine serum (FBS). MCF7/IGF2 cells were generated as previously described (7). MCF7 KO-IGF1R (MCF7^IGF1R-ve^) and KO-DDR1 (MCF7^DDR1-ve^) and the control MCF7^Cas9^ cells were purchased from Applied Biological Materials (Richmond, BC, Canada). 

### 2.3. Generation of Lentiviral Vector pLEXG418–hIRA–FLAG Lentiviral Vectors

pLEX–pCMV–IRES–PAC (PAC: puromycin-N-acetyltransferase gene; a puromycin resistance gene) lentiviral vector with cytomegalovirus constitutive promoter (pCMV) was used to obtain pLEX–pCMV–IRES–G418 (G418: geneticin resistance gene), as previously described [8]. Briefly, pLEX–pCMV–IRES–PAC was subjected to enzymatic digestion by NotI and HpaI to replace IRES–PAC with IRES–G418 sequence obtaining pLEX–pCMV–IRES–G418 (pLEXG418–EV). To generate pLEXG418–hIRA–FLAG lentiviral vectors, we amplified IR-A (hIRA v2, GENE ID: NM_001079817) and cDNA from RG215257-hIR-A plasmid (all from OriGene, Rockville, MD, USA) with the following primers containing FLAG sequence in the reverse primer: forward (Fw) hIRA: 5 0-GGACTAGTGCCACCATGGCCACCGGGGGCCG-30; reverse (Rv) hIRA: 5 0-CGACGCGTTTACTTATCGTCGTCATCCTTGTAATCGGAAGGATTGGACCGAGGC-30. hIRA–FLAG was cloned in pLEXG418–EV in SpeI and MluI restriction sites. 

### 2.4. Generation of MCF7^IGF1R-ve^/IR-A Cells

We used MCF7-KO-IGF1R (MCF7^IGF1R-ve^) for lentiviral transduction and generation of MCF7^IGF1R-ve^/IR-A-FLAG, as previously described [8]. Recombinant lentiviruses were produced by transient transfection in T Large Antigen (TLA)-HEK-293t using the calcium phosphate method according to the protocol provided by Dharmacon. The lentiviruses supernatant was concentrated and then titrated using Lenti-X p24 Rapid Titer Kit (TakaraBio, Shiga, Japan) according to the manufacturer’s protocol. MCF7^IGF1R-ve^ cells were lentivirally transduced with a multiplicity of infection (MOI) by spinoculation at 1200 *g*, 32 °C for 90 min in the presence of 8 µg/mL of polybrene. Fresh medium was replaced and cells cultivated for additional 48 h. Since MCF7^IGF1R-ve^ cells were already resistant to puromycin, we modified pLEX–pCMV–IRES–PAC lentiviral vector to obtain pLEX–pCMV–IRES–G418, as previously described [8]. 

### 2.5. Gene Silencing by siRNAs

For siRNA experiments, cells were transiently transfected with a mixture containing Opti-MEM, Lipofectamine RNAiMax, and either a pool of four scramble siRNA oligos (10 nM) or a pool of four specific siRNA oligos for DDR1 (10 nM). The specific silencer Select Pre-designed pool of four siRNA oligos for DDR1 (Human DDR1 siGENOME SMARTpool Cat M-003111-04) and the negative control, consisting of a pool of four scramble siRNAs, were from Thermo Fisher Scientific Dharmacon (Waltham, MA, USA).

### 2.6. Measurement of IGF2 in Conditioned Medium from MCF7/IGF2 and MCF7/EV Cells

The conditioned medium (CM) from MCF7/IGF2 cells and MCF7/EV was obtained by incubating cells in a serum-free medium for 48 h. Media were collected and centrifuged at 3500 rpm for 5 min to remove cell debris. Thereafter, the supernatants were collected and concentrated (75×) at 4000 *g* for 30 min using the centrifuge filters AMICON ULTRA 15 mL NMWL (Millipore, Bedford, MA, USA). Release of myc tagged IGF2 in CM was then assessed by Western blot using an anti-myc antibody. IGF2 in CM was also measured by a specific ELISA (Mediagnost, Reutlingen, Germany). The biological ability of IGF2 released in the medium was assessed by incubating wild-type MCF7 cells with CM from MCF7/IGF2 and then measuring IR/IGF1R phosphorylation by Western blot with anti-pIR/IGF1R antibodies (19H7, Santa Cruz Biotechnology, Santa Cruz, CA, USA). 

### 2.7. Collagen Stimulation and Pervanadate Treatment 

Rat tail collagen type I (Sigma-Aldrich, Saint Louis, MO, USA) and type IV (Sigma-Aldrich, Saint Louis, MO, USA) were used to stimulate cells at a final concentration of 20 µg/mL. Pervanadate (Sigma-Aldrich, Saint Louis, MO, USA) was prepared freshly, as previously described [24].

### 2.8. Immunoprecipitation and Western Blot Analysis

After collagen stimulation, cells were lysed at 4 °C in radioimmune precipitation buffer (RIPA, Sigma Aldrich, Saint Louis, MO, USA) containing phosphatase inhibitors (sodium orthovanadate 1 mM and sodium fluoride 2 mM) and proteinase inhibitors (Roche, Sigma-Aldrich, Saint Louis, MO, USA). Lysates were clarified by centrifugation (14,000 rpm) for 15 min at 4 °C, and immunoprecipitation was carried out for 18 h. The immune complexes bound to protein A Sepharose were washed twice in lysis buffer and subjected to Western blot analysis. Subconfluent cells were solubilized in lysis buffer (Tris-HCl pH 7.4 50 mM, NP-40 0.50%, EGTA 1 mM, NaCl 150 mM) for the indicated time points. To evaluate IGF2-dependent activation, cells were serum-starved for 48 h and cell lysates were subjected to Western blot analysis as previously described [8]. 

The following antibodies were used: anti-IR (C-19, sc-711) anti MCT1 (H-1) (sc-364501) (Santa Cruz Biotechnology, Santa Cruz, CA, USA), anti-phospho(p)IGF1R (Tyr1135/1136)/pIR (Tyr1150/1151) (19H7), anti-pIR (Y1334) (Invitrogen), anti-IGF1R, anti-pTyr100, anti EK2 (C64E5), and anti Myc (9B11) (Cell Signaling Technology Inc., Beverly, MA, USA); anti-IGF1R (Ab-1, aIR3) (Calbiochem, Darmstadt, Germany); anti phosphotyrosine antibody (4G10) (Upstate, Biotechnology); anti-βactin (Sigma Aldrich, Saint Louis MO, USA); anti-lactate dehydrogenase A (LDHA) (216-228, SAB1100050), anti-pyruvate kinase M2 (PKM2) (isoform M1, SAB4200094), and anti MCT4 (A304-439A) (ThermoFisher Scientific, Inc., Waltham, MA, USA); anti ARALAR, Anti-SLC25A12 (E-AB-63584), and anti NRF-1 (E-AB-16661) (Elabscience, Houston, TX, USA). Total OXPHOS human WB Antibody Cocktail (ab110411) was obtained by Abcam (Cambridge, UK). 

### 2.9. Real-Time PCR 

Total cellular RNA was extracted using TRIzol Reagent according to the manufacturer’s protocol. qRT-PCR was used to confirm the expression levels of mRNAs. Total RNA (2μg) was reverse transcribed using the ThermoScript RT (Invitrogen, Carlsbad, CA, USA) and oligo (dT) primers. Synthesized cDNA was combined in a qRT-PCR reaction using primers for the gene of interest (Table 1). 

Real-time PCR was performed with an ABI 7500 Real-Time PCR System (Applied Biosystems) using tested samples, primer sets, and SYBR Green reagent. In preliminary studies, GAPDH and S9 were compared for their ability to be used as reference genes. The results were analyzed using RefFinder (https://www.heartcure.com.au/reffinder/), a web-based tool that allows the comparison of candidate reference genes. This analysis indicated GAPDH as the most stable gene (comprehensive gene stability 1.189 vs. 1.414); therefore, in subsequent experiments, human GAPDH was used for normalization in SYBR Green chemistry. mRNA quantification was performed using the comparative CT method (ΔΔCt). 

### 2.10. Metabolic Profiling by Seahorse Assays

Cells were seeded into XFp cell culture microplates (Seahorse Biosciences, Santa Clara, CA, USA) at a density of 6000–8000 cells per well. Then, cells were treated with ligands, as indicated, and subjected to ATP Rate Assay using an XFp Extracellular Flux Analyzer (Seahorse Biosciences, Santa Clara, CA, USA) according to the manufacturer’s instructions. The ATP Rate Assay measures OXPHOS and glycolysis’s contribution to ATP production. This assay was performed under standard conditions and after 2 h of glucose deprivation. OCR and acidification were measured before and after sequential injection of 3 µM oligomycin A and 0.5 µM rotenone/antimycin A. Values were normalized to cell number.

### 2.11. Fluorescence Microscopy

Cells were seeded onto glass coverslip in 6-well plates at a density of 50% and stained with Mitotracker Orange (400 nM). Cell staining was performed using an Olympus microscope. Images were recorded using a 40× magnification under oil immersion, digitally acquired using an AxioCam ICm1 (Carl Zeiss Meditec, Jena, Germany), and processed using NIH ImageJ software, version 1.53, May 2021 (National Institutes of Health, Bethesda, MD, USA). Quantification of images was performed as follows. A total of 180 cells per condition was analyzed using the NIH ImageJ, calculating the integrated density of each cell. 

### 2.12. Densitometric and Statistical Analysis

Densitometry results were obtained by using Image Studio Lite software, version 5.2.5, 2020 (LI-COR Biosciences, Lincoln, NE, USA). Data distribution was assessed with the Shapiro–Wilk test for normality and with the asymmetry/kurtosis tests for normality. The differences between the means were evaluated by Student’s *t*-test for paired samples provided the data had normal distribution and by the Mann–Whitney test for data with non-normal data distribution. The level of significance was set at *p* < 0.05. Statistical analysis was performed with GraphPad Prism8 software, version 8.1.1, 2019 (GraphPad Software, San Diego, CA, USA). Data were expressed as means ± SEM.

## 3. Results

### 3.1. DDR1 Affects the Metabolic Reprogramming of BC Cells Constitutively Overexpressing IGF2

**Figure 1:**
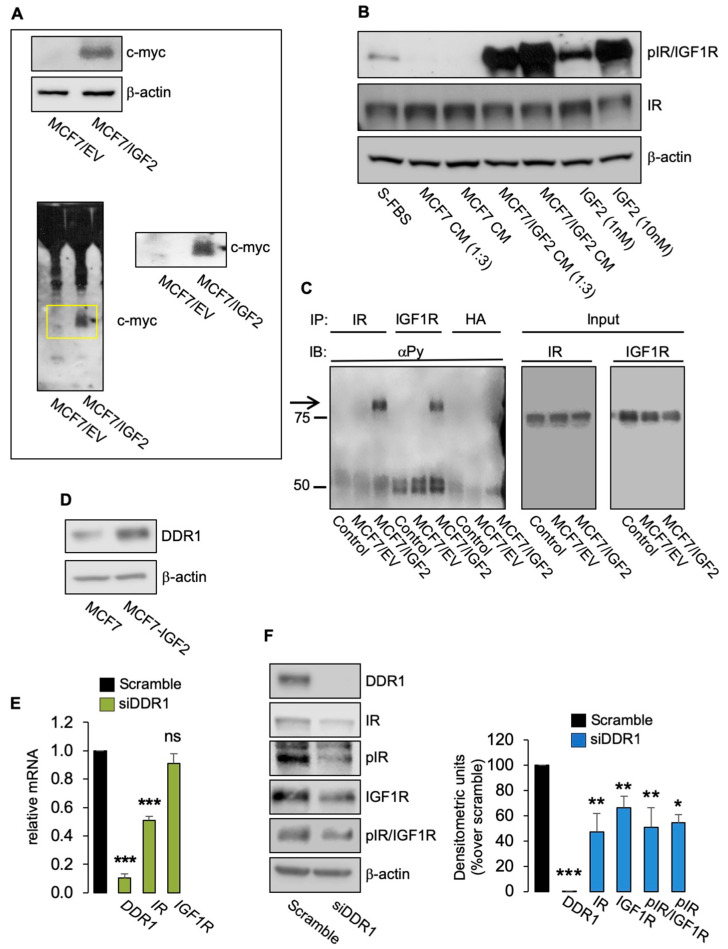
(**A**) IGF2 (myc-tagged) expression in cell lysates (upper panel) and in CM (lower panel) from MCF7/IGF2 and MCF7 EV cells by Western blot analysis. The yellow box shows the band corresponding to the IGF2 myc-tagged enlarged on the right. A representative blot of three independent experiments is shown. (**B**) CM derived from MCF7/IGF2, but not from MCF7 EV, was able to induce phosphorylation of IR/IGF1R in wild-type MCF7 cells. A representative blot of three experiments is shown. (**C**) IRs and IGF1Rs from MCF7/IGF2 and MCF7 EV cells were immunoprecipitated with specific antibodies and probed with anti-PY (4G10), as described in Methods. Total lysates (input) were evaluated as control and blotted with anti-IR and anti-IGF1R. (**D**) MCF7/IGF2 cells showing increased DDR1 protein expression compared to parental MCF7 cells. A representative blot of three experiments is shown. (**E**) Expression of DDR1, IR, and IGF1R mRNAs in MCF7/IGF2 cells transiently transfected with siRNA to DDR1 or scramble siRNAs, as measured by qRT-PCR. GAPDH was used as housekeeping control gene. Data are presented as the means ± SEM of three independent experiments. (**F**) MCF7/IGF2 cells transfected with siDDR1 or scramble oligonucleotides were subjected to immunoblot analysis with the indicated primary antibodies. In particular, to assess receptor autophosphorylation, we used anti-phospho-(p)IGF1R (Tyr1135/1136)/pIR (Tyr1150/1151) antibody detecting both pIR and pIGF1R and anti-pIR (Tyr1334) antibody, specific for pIR. *β*-actin was used as control for protein loading. Blots are representative of three independent experiments. Side panels show densitometric analysis. (ns, not significant; *, *p* < 0.05; **, *p* < 0.01; ***, *p* < 0.001; Student’s *t*-test).

To investigate whether DDR1 affected IIGFs-driven metabolic reprogramming, we used human BC cells MCF7 stably transfected with an myc-tagged IGF2 vector (MCF7/IGF2) [8]. We have previously shown that MCF7/IGF2 cells are characterized by constitutive activation of both the IGF1R and the IR-A, phosphorylation of key downstream signaling molecules such as AKT and ERK1/2, and increased metabolic activity sustained not only by high aerobic glycolysis but also high oxidative phosphorylation (OxPhos) [8]. To further characterize MCF7/IGF2 cells, we asked whether they actually secrete biologically active IGF2 in the conditioned medium (CM). Indeed, myc tagged IGF2 was not only present in cell lysates (Figure 1A, upper panel) but was also released in CM (Figure 1A, lower panel) and found to be able to phosphorylate IR/IGF1R in wild-type MCF7 cells (Figure 1B). Selective immunoprecipitation of IR and IGF1R further demonstrated that both receptors are constitutively phosphorylated in MCF7/IGF2 cells but not in MCF7 EV (Figure 1C). Finally, IGF2 protein concentration in CM was measured by ELISA. IGF2 concentration was >100 ng/mL in CM from MCF7/IGF2 cells and 1.8 ± 0.05 ng/mL in CM from MCF7/EV.

As shown in Figure 1D, MCF7/IGF2 cells expressed high DDR1 levels. We first knocked down DDR1 in MCF7/IGF2 cells with a pool of four specific small interfering RNA (siRNA) oligos against DDR1 (siDDR1). In agreement with previous results [9], DDR1-depleted cells showed reduced mRNA expression levels of IR as compared with scrambled transfected cells (Figure 1E), while IGF1R mRNA levels were unaffected (Figure 1E). However, both IR and IGF-IR protein levels significantly decreased, as shown in Figure 1F. MCF7/IGF2 cells showed constitutive phosphorylation of IR/IGF1R and specific IR phosphorylation as detected by phosphoantibody against Tyr1334. Both phosphorylation signals were reduced by DDR1 silencing (Figure 1F) according to reduced receptor levels. 

**Figure 2:**
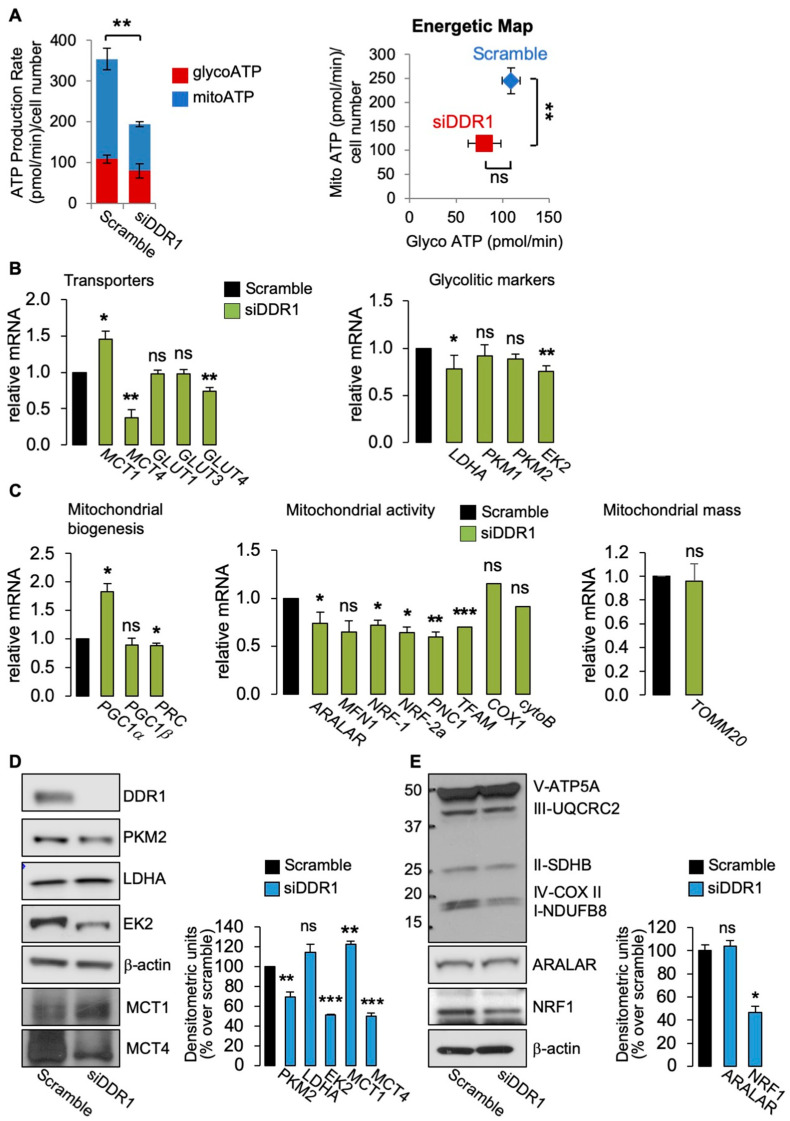
(**A**) ATP production rate in MCF7/IGF2 cells silenced for DDR1 and in control cells treated with scramble siRNAs. Glycolytic ATP (red columns-glycoATP) and the mitochondrial (blue columns—mitoATP) production rates were evaluated according to the manufacturer’s instructions (Agilent ATP test). OCR and ECAR were first measured in basal conditions. Injection of oligomycin resulted in inhibition of mitochondrial ATP synthesis and decrease in OCR, allowing the mitoATP production rate to be quantified. Complete inhibition of mitochondrial respiration with rotenone plus antimycin A accounting for mitochondrial-associated acidification allowed the calculation of the glycoATP production rate. The presented histogram (left panel) and the energetic map (right panel) show the mean and range from three independent experiments. (**B**) Glycolysis related markers: MCF7/IGF2 cells transiently transfected with an siRNA for DDR1 (green columns) or scramble siRNAs (black columns) were processed for mRNA expression of glycolysis-related transporters: MCT1, MCT4, GLUT1-4 (left panel), and enzymes LDHA, PKM1, PKM2, and EK2 (right panel). (**C**) OxPhos related markers in MCF7/IGF2 cells silenced for DDR1 (green columns) or treated with scramble siRNAs (black columns). Markers of mitochondrial biogenesis (PGC1α, PGC1β, PRC) (left panel), and mitochondrial markers (ARALAR, MFN1, NRF-1, NRF-2a, TFAM, and PNC1) (middle panel) were measured by qRT-PCR analysis. Mitochondrial activity was evaluated by mRNA expression levels of COX1 and cytoB (middle panel), and mitochondrial mass was assessed by measuring mRNA levels of mitochondrial outer membrane protein (TOMM20) (right panel). GAPDH was used as housekeeping control gene. Values are expressed as the means ± SEM of three separate experiments. (**D**) Western blot of selected glycolysis related molecules (PKM2, LDHA, MCT1, MCT4, and EK2) in MCF7/IGF2 cells silenced for DDR1 or treated with scramble oligonucleotides (left panel) and densitometric analysis of results obtained in three independent experiments (right panel). (**E**) Western blot with OxPhos antibodies cocktail against mitochondrial complexes showed a decrease of mitochondrial complex IV and I. NRF1 protein was also markedly decreased, whereas ARALAR did not show significant changes. Graphs represent the mean ± SEM of densitometric analysis of three independent experiments, where values were normalized to β-actin. (ns, not significant; *, *p* < 0.05; **, *p* < 0.01; ***, *p* < 0.001; Student’s *t*-test).

We then evaluated the effect of DDR1 depletion in MCF7/IGF2 bioenergetics by assessing total ATP production as well as glycolysis–derived ATP (glycoATP) and mitochondrial OxPhos–derived ATP production (mitoATP) using the Seahorse technology. The cellular rate of ATP production is a highly informative measure of cellular energy demands required for cellular functions. Notably, in DDR1-silenced MCF7/IGF2 cells, total ATP production was significantly decreased as compared to control cells (Figure 2A, left graph) with mitoATP production being more affected than glycoATP production rate (Figure 2A, right panel).

Glucose uptake is mediated by membrane-bound glucose transporters (GLUTs) and expression of GLUTs is often increased in various glycolytic cancers including BC [25,26]. As mentioned, glycolysis produces substantial quantities of lactate. However, in cancer, intracellular lactic acidosis, which would promote cell death, is prevented by extracellular lactate transport through proton-linked monocarboxylate transporters (MCTs), which act as important regulators of intracellular pH [27]. Notably, acidification of the extracellular microenvironment is associated with tumor progression [28]. Therefore, we evaluated the expression of GLUT1-4, MCT1 (which transports lactate within the cell), and MCT4 (which controls cellular lactate efflux). Consistent with the reduced ATP production, MCF7/IGF2 cells silenced for DDR1 showed reduced GLUT4 and MCT4 mRNA levels (Figure 2B, left panel) and reduced mRNAs expression levels of the glycolytic enzymes hexokinase 2 (EK2) and LDHA (Figure 2B, right panel) as compared to control cells. 

**Figure 3:**
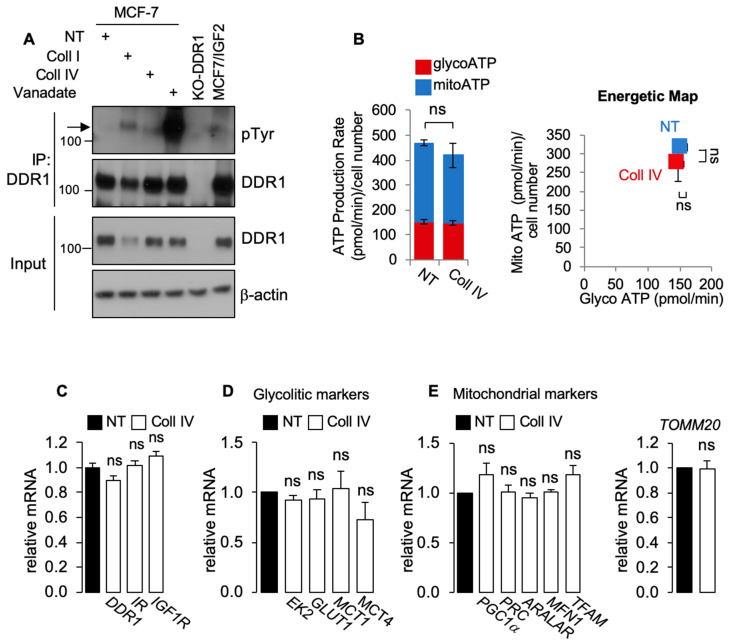
(**A**) MCF7 cells were exposed to 20 μg/mL of collagen I (Coll I) or collagen IV (Coll IV) for 6 h, and to 1mM of Na3VO4 (Van) for 90 min as positive control for DDR1 phosphorylation. Untreated cells were indicated as NT (not treated). DDR1-KO cells were used as negative control. Cells were then solubilized and lysates immunoprecipitated with an anti-DDR1 antibody and analyzed by immunoblot. Total lysates (input) were evaluated as control. Filters were probed with anti-DDR1 and anti-PY antibodies, as indicated. A representative blot of three independent experiments is shown. (**B**) ATP production rate (left panel) and energetic map (right panel) in MCF7/IGF2 cells exposed or not to collagen IV (Coll IV) at a concentration of 20 µg/mL for 48 h. Values are expressed as means ± SEM of three separate experiments. (**C**) MCF7/IGF2 cells were exposed or not to collagen IV (Coll IV) at a concentration of 20 µg/mL for 48 h and processed to evaluate mRNA expression for DDR1, IR, and IGF1R. Normalization was done using human GAPDH as a housekeeping control gene. Values are expressed as means ± SEM of three independent experiments. (**D**) MCF7/IGF2 cells treated with collagen IV (white columns) or untreated (NT, black columns) were processed to evaluate mRNA expression for glycolysis related enzyme (EK2) and transporters (GLUT1, MCT1, and MCT4). (**E**) OxPhos-related markers were measured in MCF7/IGF2 cells, treated or not treated (NT) with collagen IV. Mitochondrial markers (PGC1α, PRC, ARALAR, MFN1, and TFAM) were measured by qRT-PCR (left panel) using GAPDH as a housekeeping control gene. TOMM20 was used as a surrogate marker for mitochondrial mass (right panel). Values are expressed as means ± SEM of three separate experiments. (ns—not significant; Student’s *t*-test).

Moreover, in MCF7/IGF2 cells silenced for DDR1, we observed changes in mRNA levels for markers of mitochondria biogenesis and activity and mitochondrial mass (Figure 3C). Specifically, we found increased gene expression of the peroxisome proliferator-activated receptor gamma coactivator-1α (PGC1α), while PGC1β expression was unaffected. Both are key members of the family of transcriptional co-activators, involved in mitochondrial biogenesis [23]. In contrast, the expression of PGC1α−related coactivator (PRC) was significantly reduced in DDR1-silenced cells compared to control cells (Figure 2C, left panel). We also observed decreased gene expression of aspartate–glutamate mitochondrial carrier 1 (ARALAR/AGC1) and nuclear respiratory factor 1 and 2 alpha (NRF-1 and NRF-2a), nuclear-encoded mitochondrial carrier proteins. Mitochondrial transcription factor A (TFAM), encoding for a key activator of mitochondrial transcription, and a mitochondrial pyrimidine nucleotide carrier, PNC1), encoding for a protein essential for mitochondria maintenance, were also reduced in DDR1-silenced cells. We also observed a trend in reduced expression levels of mitofusin-1 (MFN1) encoding for a protein involved in mitochondrial fusion (Figure 2C, middle panel), of cytochrome C oxidase 1 (COX1), one of the three mtDNA encoded subunits of the respiratory complex IV (Figure 2C, middle panel), and of the translocase of outer membrane (TOMM20) (Figure 2C, right panel) in DDR1-silenced MCF7/IGF2 compared to control cells. These differences, however, did not reach statistical significance. We then measured the protein expression of a panel of key metabolism-related molecules in MCF7/IGF2 cells before and after DDR1 silencing. The results obtained indicated that DDR1 silencing was associated with upregulation of MCT1 protein along with downregulation of MCT4, PKM2, and EK2 protein expression (Figure 2D). Furthermore, DDR1 silencing was concomitantly associated with downregulation of mitochondrial complex IV and I and NRF1, while ARALAR/AGC1 did not vary significantly (Figure 2E).

**Figure 4:**
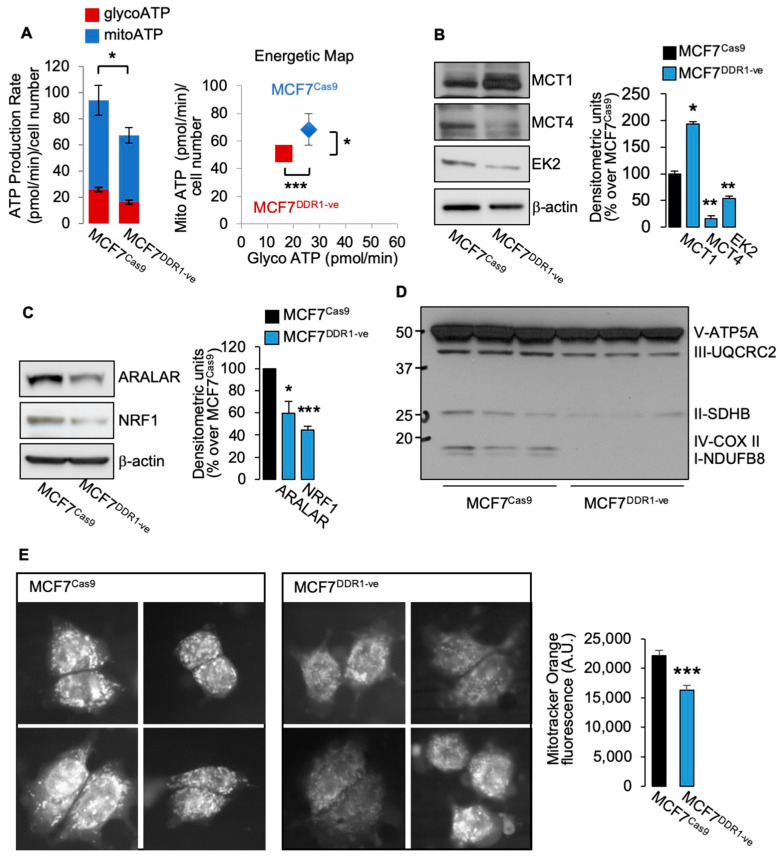
(**A**) MCF7^DDR1-ve^ cells and parental (MCF7^CAS9^ cells were subjected to real time cell bioenergetics (Agilent ATP test). Values are means ± SEM of three separate experiments, which are compared with the Student’s *t*-test. (**B**–**D**) The same cells as in (A) were grown in 10% FBS, lysed, and analyzed by SDS-PAGE and immunoblot for the indicated proteins. β-actin antibody was used as control for protein loading. A representative blot of three independent experiments is shown. Graphs show the mean ± SEM of densitometric analysis of three independent experiments that are compared with the Student’s *t*-test. (**E**) Mitochondrial activity was evaluated in MCF7^DDR1-ve^ cells and parental MCF7^CAS9^ by immunofluorescence using the probe MitoTracker Orange. Four representative images are shown for each cell line. Graphs represent the mean ± SEM of densitometric analysis of three independent experiments that are compared with the Mann–Whitney test. (ns—not significant; * *p* < 0.05; ** *p* < 0.01; *** *p* < 0.001).

Next, we asked whether DDR1 phosphorylation induced after collagen stimulation might affect cell metabolism and/or IIGFs receptor expression. Incubation of MCF-7 cells with collagen IV (20 µg/mL for 48 h) induced DDR1 phosphorylation (Figure 3A), which was not associated with significant changes in mRNA expression levels of DDR1, IR, or IGF1R (Figure 3B). Notably, incubation with collagen IV did not affect ATP production (Figure 3C), glycolytic (Figure 4D), or OxPhos (Figure 3E) markers, further indicating that the metabolic changes induced by DDR1 silencing are independent from DDR1 collagen-binding activity.

### 3.2. MCF7^DDR1-ve^ Cells Show Impaired Metabolic Activity

Moreover, we studied several metabolic parameters in MCF7^DDR1-ve^ cells as compared to control MCF7 Cas9 cells. MCF7^DDR1-ve^ cells revealed impaired ATP production (Figure 4A), and significant changes in protein expression of key metabolic regulators, including upregulation of MCT1 and downregulation of MCT4 and hexokinase 2 (Figure 4B). Moreover, molecules involved in OxPhos were also markedly reduced, including NRF1 and ARALAR (Figure 4C) and mitochondrial complexes (Figure 4D). When cells were stained with mitotracker Orange, which is related to the mitochondrial membrane potential and mitochondrial activity, MCF7^DDR1-ve^ cells showed a significantly lower staining intensity (Figure 4E). Together, these findings indicate that MCF7 cells stably lacking DDR1 have impaired glucose metabolism and ATP production.

### 3.3. DDR1 Silencing Decreases the Metabolic Activity of BC Cells Overexpressing IR-A

**Figure 5:**
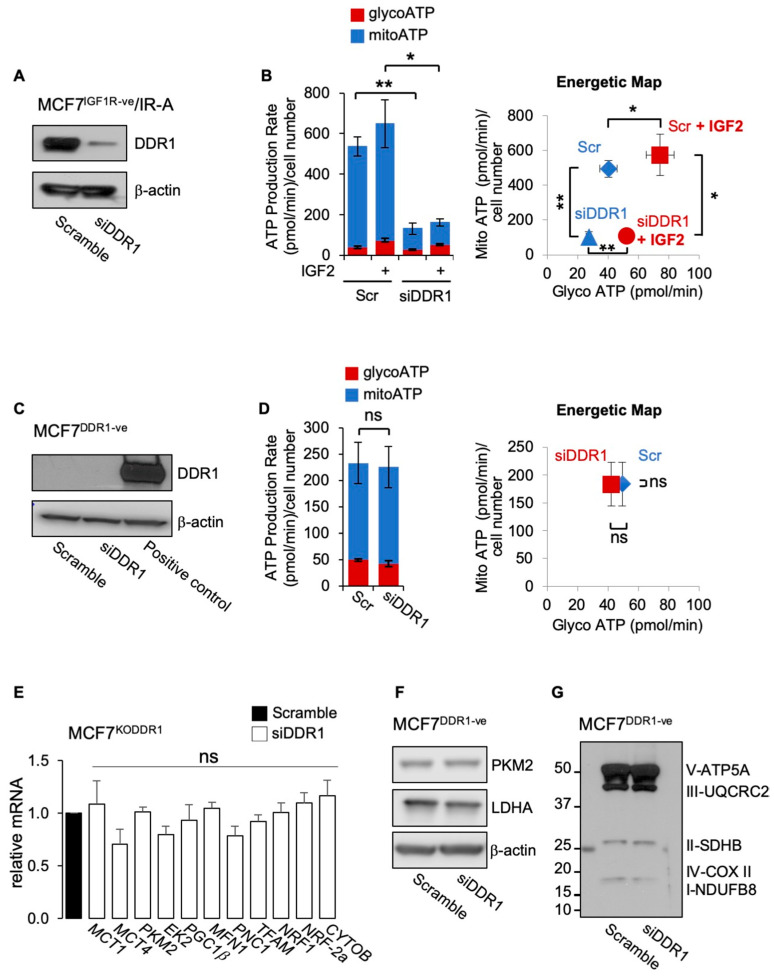
(**A**) MCF7^IGF1R-ve^/IR-A cells with or without DDR1 silencing were analyzed by Western immunoblotting for DDR1 expression using polyclonal antibodies against the C-terminus of DDR1, as indicated. β-actin antibody was used as control for protein loading. A representative blot of three independent experiments is shown. (**B**) ATP production rate in MCF7^IGF1R-ve^/IR-A cells silenced for DDR1 and in control cells treated with scramble siRNAs, upon stimulation with 100 nM IGF2 for 48 h. Glycolytic ATP (red columns—glycoATP) and mitochondrial ATP (blue columns—mitoATP) production rates were evaluated according to the manufacturer’s instructions as described above (Agilent ATP test). The presented histogram (left panel) and the energetic map (right panel) show the mean and range from three independent experiments. (**C**) MCF7^DDR1-ve^ cells were analyzed by Western immunoblot for DDR1 expression using polyclonal antibodies against the C-terminus of DDR1, as indicated. A positive control is also shown. β-actin antibody was used as control for protein loading. A representative blot of three independent experiments is shown. (**D**) ATP production rate in MCF7^DDR1-ve^ silenced for DDR1 and in control cells treated with scramble siRNAs. The presented histogram (left panel) and the energetic map (right panel) show the mean and range from three independent experiments. (**E**) MCF7^DDR1-ve^ cells and parental MCF7^Cas9^ control cells grown in complete medium were treated with siDDR1 or scramble siRNAs and analyzed for mRNA expression of transporters (MCT1, MCT4), glycolysis related enzymes (PKM2, EK2), and mitochondrial markers (PGC1β, MFN1, PNC1, TFAM, NRF1, NRF-2a, and CYTOB). Normalization was performed using human GAPDH as a housekeeping control gene. Values are expressed as means ± SEM of three independent experiments. (**F**–**G**) MCF7^DDR1-ve^ and MCF7^Cas9^ cells silenced or not for DDR1 were analyzed by SDS-PAGE and immunoblot for the indicated proteins. A representative blot of three independent experiments is shown. (ns—not significant; *, *p* < 0.05; **, *p* < 0.01; Student’s *t*-test).

We then investigated whether DDR1 silencing could inhibit the metabolic activity of BC cells that overexpress the oncofetal IR-A, a frequent occurrence in BC. To avoid the possible interfering activity of IGF1R, we employed MCF7 cells stably knocked-out for the *IGF1R* gene (MCF7^IGF1R-ve^) and overexpressing the IR-A (MCF7^IGF1R-ve^/IR-A). As shown in Figure 5A, DDR1 silencing significantly inhibited ATP production both in the presence and in the absence of IGF2 stimulation (Figure 5B). The inhibition was especially evident on the mitochondrial ATP production rate in samples without DDR1 (Figure 5B, left panel). To additionally demonstrate the specific DDR1 action in the inhibition of ATP production, we performed control experiments in MCF7 cells stably knocked-out for DDR1 (MCF7^DDR1-ve^ cells) and therefore not expressing detectable levels of DDR1 protein in Western blot analysis (Figure 5C). As expected, transfection with siRNA against DDR1 did not affect ATP production rate in these cells (Figure 5D) or the expression of metabolic-related genes (Figure 5E). Moreover, it did not affect protein expression of PKM2, LDHA (Figure 5F), or mitochondrial complexes (Figure 5G).

### 3.4. The Metabolic Action of DDR1 Also Occurs Independently of IR/IGF1R Phosphorylation

**Figure 6:**
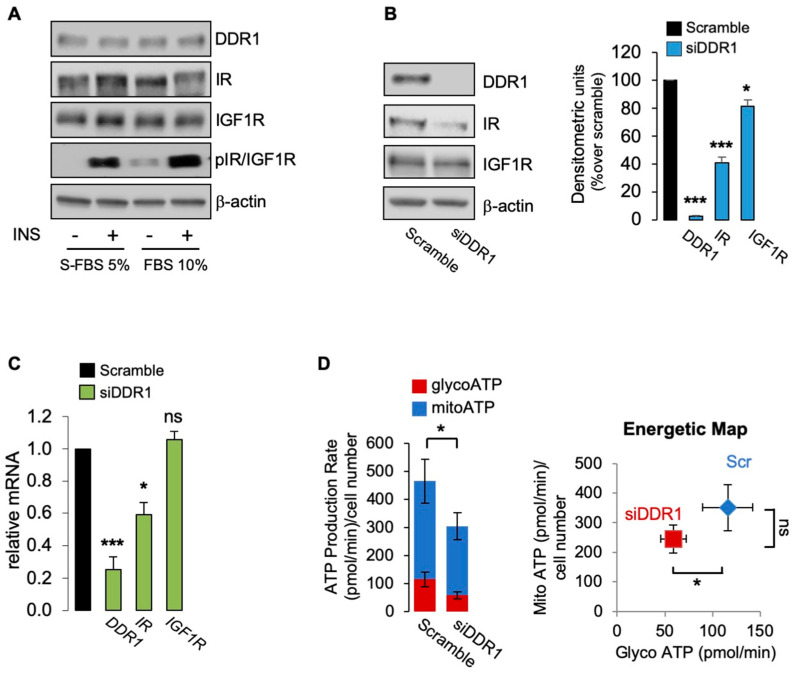
(**A**) MCF7 cells grown in 5% charcoal stripped fetal bovine serum (S-FBS) and in 10% FBS were stimulated with vehicle (-) or insulin (10 nM for 5 min), and IR/IGF1R phosphorylation evaluated by Western blotting analysis using anti-phospho-(p)IGF1R (Tyr1135/1136)/pIR (Tyr1150/1151) antibody. β-actin was used as control for protein loading. Blots are representative of three independent experiments. The histograms represent the mean ± SEM of densitometric analysis of three independent experiments; (**B**) MCF7 cells cultured in S-FBS were transiently transfected with siRNA to DDR1 or scramble siRNAs. After 48 h, cells were lysed and analyzed by SDS-PAGE and immunoblotted with the indicated primary antibodies. β-actin was used as control for protein loading. Blots are representative of three independent experiments. The histogram represents the mean ± SEM of densitometric analysis of three independent experiments. (**C**) Cells cultures as in (**B**) were analyzed for DDR1, IR, and IGF1R mRNAs expression by qRT-PCR analysis. Normalization was done using human GAPDH as housekeeping control genes. Data are presented as the mean ± SEM (error bars) from three independent experiments; (**D**) ATP production rate in MCF7 cultured in 5% S-FBS silenced for DDR1 and in control cells treated with scramble siRNAs. The presented histogram (left panel) and the energetic map (right panel) show the mean and range from three independent experiments. (ns—not significant; *, *p* < 0.05; ***, *p* < 0.001).

Having demonstrated that the effect of DDR1 silencing is specific, independent of collagen-binding activity, and does not require IGF1R expression, we additionally investigated whether it might occur in the absence of IR/IGF1R phosphorylation [9,12]. Thus, we cultured MCF7 cells in 10% FBS or in 5% charcoal-stripped fetal bovine serum (S-FBS) and evaluated IR/IGF1R phosphorylation using specific phosphoantibodies in cells stimulated or not with insulin (10 nM for 5 min). As shown in Figure 5A, basal IR/IGF1R tyrosine phosphorylation was very weak in cells cultured in 10% FBS and undetectable in S-FBS cultures. Insulin stimulation elicited a strong IR/IGF1R phosphorylation in both conditions. Using unstimulated MCF7 cells cultured in S-FBS, we confirmed that DDR1 silencing induces a significant decrease of IR and IGF1R protein expression (Figure 6B). As expected, IR mRNA but nor IGF1R mRNA also decreased following DDR1 silencing (Figure 6C). In these cells, which lack IR/IGF1R phosphorylation, DDR1 silencing was still associated with cell ATP production, mainly at the expenses of mitoATP (Figure 6D), a finding resembling what was observed in MCF7/IGF2 cells that are instead characterized by high constitutive IR/IGF1R activation (see Figure 1B). Comparable results were also obtained in unstimulated MCF7 cells cultured in 10% FCS that show minimal IR/IGF1R phosphorylation (not shown). Collectively, these results indicate that metabolic reprogramming induced by DDR1 silencing in MCF7 cells may occur also in unstimulated MCF-7 lacking IR/IGF1R phosphorylation, and therefore, independently of IIGFs activation.

## 4. Discussion

We previously discovered that upregulation of the IR-A and constitutive IGF2 expression represent novel non-mutational mechanisms contributing to the metabolic reprogramming and increased metabolic flexibility of human BC cells [8]. Moreover, insulin stimulation elicited similar metabolic effects to IGF2 in BC cells overexpressing IR-A also in the absence of IGF1R, indicating that the metabolic action of IR-A may not require a functional IGF1R [8]. These previous findings are in line with the concept that hyperinsulinemia of obese and/or diabetic patients may contribute to metabolic reprogramming and tumor promotion of BC overexpressing IR-A. As no pharmacological approach is available to specifically target the IR-A in cancer [29], we evaluated whether DDR1 targeting could be alternatively used as a strategy to inhibit IR-A-driven metabolic reprogramming in BC cells.

We initially used MCF7/IGF2 cells as a model system. As previously shown, MCF7/IGF2 cells are characterized by constitutive activation of IR-A and IGF1R; downstream signaling cascades; enhanced ability to proliferate, migrate, and form colonies; and increased metabolic activity (both in basal conditions and under increased energy demand) [8]. Importantly, in MCF7/IGF2 cells, DDR1 was upregulated, a finding consistent with the previously described feed-forward loop linking DDR1 and IIGFs [9,10,13]. Specifically, we previously demonstrated that IIGFs’ activation upregulates DDR1 expression by suppressing miR-199a-5p through the PI3K/AKT pathway [14]. Here, we discovered that in MCF7/IGF2 cells, DDR1 silencing reduced ATP production to approximately 50% of the baseline. Both glycoATP and mitoATP were inhibited, with the latter being relatively more affected. Consistently, DDR1 silencing was associated with a decrease of glucose transporter GLUT4, of lactate transporter MCT4, which controls lactate cellular efflux, and of glycolytic enzymes, such as hexokinase and PKM2. Concomitantly, we observed reduced gene expression of mitochondrial biogenesis and activity markers, including PRC, a key regulator of mitochondrial biogenesis and ATP production [30], ARALAR/AGC1, involved in aspartate transport from the mitochondrion and regeneration of cytosolic glutathione [31], and NRF-1 and NRF-2a, two PGC-1α- regulated transcription factors involved in the regulation of OxPhos related genes [32]. The expression of a panel of key metabolism-related molecules, including MCT1, MCT4, hexokinase 2, PKM2, NRF-1, and mitochondrial complexes was similarly modulated by DDR1 silencing. All these factors play a key role in cancer development and progression [32,33,34]. DDR1 silencing was associated with downregulation of both IR and IGF1R proteins and reduction of IR/IGF1R phosphorylation, suggesting that the metabolic impact of DDR1 on MCF7/IGF2 cells may be partially mediated by its ability to modulate IR-A and IGF1R expression and affect the activation of downstream signaling cascades including the PI3K/AKT and ERK1/2 cascades [8]. In agreement with these findings, we previously discovered that DDR1 silencing inhibited several insulin/IGFs-mediated responses, such as cell proliferation, migration, and invasion in both ER+ and in triple-negative BC cells [9]. Moreover, in thyroid cancer cells, DDR1 silencing promoted differentiation [10]. The data presented here suggest therefore that reduced cell bioenergetics might contribute to these biological responses induced by DDR1 depletion.

Notably, cell exposure to collagen IV did not affect cell metabolism or the expression of glycolytic and mitochondrial-related markers. These findings indicate that DDR1 metabolic effects are independent of its collagen binding activity as previously shown for its functional crosstalk with IIGFs [11,13]. In line with these findings supporting the relevance of DDR1 non-canonical actions, Gao et al. recently demonstrated that DDR1’s pro-metastatic functions do not require its kinase activity in lung and breast cancer models [16]. DDR1 silencing also effectively reduced ATP production in MCF7 cells overexpressing IR-A, a cell model that mimics the IR background often found in human BCs [35]. This effect was also observed in the absence of IGF1R and of IGF2, suggesting that the metabolic effect of DDR1 silencing does not require IGF1R activation. Indeed, DDR1 silencing was associated with reduced ATP production also in wild-type MCF7 cells cultured in 5% charcoal-stripped FBS and characterized by undetectable IR/IGF1R phosphorylation. To further corroborate these data, we also show that, compared to control cells, MCF7 cells knocked out for DDR1 (MCF7^DDR1-ve^ cells) exhibit a significantly lower ATP production along with the downregulation of key molecules related to both glycolysis and OxPhos. As expected, siRNA transfection against DDR1 did not affect either cell metabolism or metabolic enzyme expression in MCF7^DDR1-ve^ cells, confirming the absence of off-target metabolic effects of DDR1 silencing. Together, our results indicate that DDR1 silencing may specifically reduce BC cell bioenergetics by multiple mechanisms that involve downregulation of IIGFs but also other non-canonical mechanisms.

Numerous studies have reported differential expression and mutations of DDR1 in several cancer types, clearly indicating that DDR1 can be considered a new player in different aspects of tumor progression [10,13], including metastatic spread, suppression of antitumor immunity [36], and modulation of cell response to chemotherapy [37,38,39]. However, to our knowledge, no specific studies have addressed DDR1 role in cancer cells metabolic reprogramming.

One limitation of our study is that it has been carried out in a single ER+ BC cell line. Indeed, there are several reasons for this choice. We have previously characterized MCF7 cells for their proliferative and invasive response to insulin and IGF2, for the expression of IR-A, IGF1R, and their crosstalk with DDR1, and for their metabolic activity in response to insulin and IGF2 [8]. Moreover, we have obtained stable MCF7/IGF2 cell clones and MCF7 knocked out for either DDR1 or IGF1R. In future experiments, we are planning to extend these observations in a more comprehensive panel of BC cell lines. Nevertheless, our data strongly suggest that DDR1 might be a useful target to address metabolic reprogramming in advanced BCs, especially in those tumors overexpressing both DDR1 and IR-A.

## 5. Conclusions

**Figure 7:**
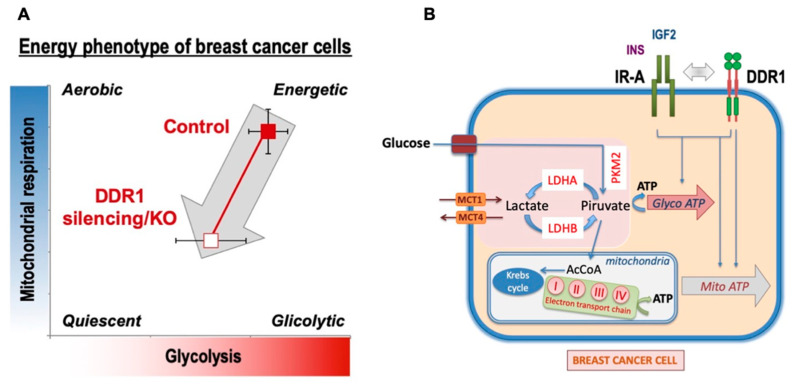
Schematic representation of the impact of DDR1 silencing/knocking out on MCF7 human breast cancer cells bioenergetics. (**A**) DDR1 silencing of MCF7 cells was accompanied by a reduction of ATP production (both glycoATP and mitoATP) also in the presence of IGF2, which is known to stimulate cell metabolism. A similar ATP reduction was observed in MCF7^DDR1-ve^ cells compared to the control cells. (**B**) Several key molecules involved in the regulation of both glycolysis and OxPhos were negatively affected by DDR1 silencing or knocking out. Together, the data support the notion that DDR1 targeting may negatively affect breast cancer cell bioenergetics at least partially by downregulating IR-A.

Overall, our data indicate that, in human BC cells, DDR1 regulates metabolic reprogramming in a collagen-independent manner and that activated IR-A and IGF1R may play a role in these effects. Notably, in BC tissues, the IR-A is frequently overexpressed and constitutively activated [40,41,42,43]. The present study identifies DDR1 as a suitable target for inhibiting the metabolic effects of IR-A overexpression in BC cells in the presence or absence of the IGF1R. However, DDR1 silencing also affected MCF7 cell metabolism independently of IR or IGF1R activation (Figure 7). The characterization of the precise mechanisms underlying DDR1 intrinsic metabolic effects requires further studies. It is worth noting that, although cancer cells preferentially metabolize glucose through aerobic glycolysis rather than using OxPhos [22], high glycolytic activity may coexist with efficient OxPhos, which is especially required for cell migration, metastasis, and resistance to TK inhibitors (TKI) [23]. DDR1 depletion affects both glycolysis and OxPhos, but especially the latter, suggesting that DDR1 targeting might preferentially affect BC dissemination and resistance to therapies.

## Figures and Tables

**Table 1:** PRC primers used.

*Gene*	*Primers*
*ARALAR*	*Fw 5′-CGAGACATTCCCTTCTCTGC-3′*
	*Rv 5′-GTCCCTTTCCAAAATGCTGA-3′*
*CytoB*	*Fw 5′-ATGGCTGAATCATCCGCT AC-3’*
	*Rv 5′-5′-GTGTGAGGGTGGGACTGTCT-3’*
*Cox1*	*Fw 5′-5′-CTCCTACTCCTGCTCGCATC-3’*
	*Rv 5′-5′-GGGTGACCGAAAAATCAGAA-3’*
*DDR1*	*Fw 5′-5′-GCGTCTGTCTGCGGGTAGAG-3’*
	*Rv 5′-5′-ACGGCCTCAGATAAATACATTGTCT-3’*
*GAPDH*	*Fw 5′-ACCCACTCCTCCACCTTTG-3′*
	*Rv 5′-CTCTTGTGCTCTTGCTGGG-3′*
*GLUT1*	*Fw 5′-ATCGTGGCCATCTTTGGCTTTGTG-3′*
	*Rv 5′-CTGGAAGCACATGCCCACAATGAA-3′*
*GLUT3*	*Fw 5′-AGCTCTCTGGGATCAATGCTGTGT-3′*
	*Rv 5′-ATGGTGGCATAGATGGGCTCTTGA-3′*
*GLUT4*	*Fw 5′-TCGTGGCCATATTTGGCTTTGTGG-3′*
	*Rv 5′-TAAGGACCCATAGCATCCGCAACA-3′*
*Hexokinase2*	*Fw 5′-TTGGCTTTTGCTTGGCAGAG-3′*
	*Rv 5′-TCTTAATAGGGCCAAGCTCAGC-3′*
*IGF1R*	*Fw 5′-5′-CGATTAACTGAGAAGAGGAGTTCG-3’*
	*Rv 5′-5′-TGGTGGAGAACGACCATATCC-3’*
*IR total*	*Fw 5′-CGTGGAGGATAATTACATCGTGTT-3′*
	*Rv 5′-TGGTCGGGCAAACTTTCTG-3′*
*LDHA*	*Fw 5′-CATGGCAGCCTTTTCCTTAG-3′*
	*Rv 5′-ATGACCAGCTTGGAGTTTGC -3′*
*MCT1*	*Fw 5′-GTGGAATGCTGTCCTGTCCTC-3′*
	*Rv 5′-TCGATAATTGATGCCCATGCC-3′*
*MCT4*	*Fw 5′-ATTGGCCTGGTGCTGCTGATG-3′*
	*Rv 5′-CGAGTCTGCAGGAGGCTTGTG-3′*
*MFN1*	*Fw 5′-TGTTTTGGTCGCAAACTCTG-3′*
	*Rv 5′-CTGTCTGCGTACGTCTTCCA-3′*
*NRF1*	*Fw 5′-CGCTCTGAGAACTTCATGGAGGAACAC-3′*
	*Rv 5′-GCCACATGGACCTGCTGCACTT-3′*
*NRF2a*	*Fw 5′-AACAAGAACGCCTTGGGATAC-3′*
	*Rv 5′-GTGAGGTCTATATCGGTCATGCT-3′*
*PGC1α*	*Fw 5′-CAAGCCAAACCAACAACTTTATCTCT-3′*
	*Rv 5′-CACACTTAAGGTGCGTTCAATAGTC-3′*
*PGC1β*	*Fw 5′-GCTCAAGCTCTGGCTCTTCA-3′*
	*Rv 5′-ATGCTTGGCGTTCTGTCTGA-3′*
*PKM1*	*Fw 5′-TGAAGAACTTGTGCGAGCCT-3′*
	*Rv 5′-GCCAGACTCCGTCAGAACTA-3′*
*PKM2*	*Fw 5′-TTACCAGCGACCCCACAGAA-3′*
	*Rv 5′-GACGATTATGGCCCCACTGC-3′*
*PNC1*	*Fw 5′-GCTCTGCAGCTTTTATCACAAATTC-3′*
	*Rv 5′-AACGTAACGAGCACACTGGAGTG-3′*
*PRC*	*Fw 5′-CAAGCAGAAACAGAAGAGAGAAG-3′*
	*Rv 5′-GGTGGGATGACAAGACAAGG-3′*
*TFAM*	*Fw 5′-ACTGCGCTCCCCCTTCAG-3′*
	*Rv 5′-ACAGATGAAAACCACCTCGGTAA-3′*
*TOMM20*	*Fw 5′-GCTGGGCTTTCCAAGTTACC-3′*
	*Rv 5′-TGTCAGATGGTCTACGCCCT-3′*

*Fw*, Forward; *Rv*, Reverse.

## References

[1] Vogel W., Gish G.D., Alves F., Pawson T. (1997). The Discoidin Domain Receptor Tyrosine Kinases Are Activated by Collagen. Mol. Cell.

[2] Abdulhussein R., McFadden C., Fuentes-Prior P., Vogel W.F. (2004). Exploring the Collagen-Binding Site of the DDR1 Tyrosine Kinase Receptor. J. Biol. Chem..

[3] Avivi-Green C., Singal M., Vogel W.F. (2006). Discoidin Domain Receptor 1–Deficient Mice Are Resistant to Bleomycin-Induced Lung Fibrosis. Am. J. Respir. Crit. Care Med..

[4] Franco C., Hou G., Ahmad P.J., Fu E.Y., Koh L., Vogel W.F., Bendeck M.P. (2008). Discoidin Domain Receptor 1 (Ddr1) Deletion Decreases Atherosclerosis by Accelerating Matrix Accumulation and Reducing Inflammation in Low-Density Lipoprotein Receptor–Deficient Mice. Circ. Res..

[5] Guerrot D., Kerroch M., Placier S., Vandermeersch S., Trivin C., Mael-Ainin M., Chatziantoniou C., Dussaule J.-C. (2011). Discoidin Domain Receptor 1 Is a Major Mediator of Inflammation and Fibrosis in Obstructive Nephropathy. Am. J. Pathol..

[6] Valiathan R.R., Marco M., Leitinger B., Kleer C.G., Fridman R. (2012). Discoidin Domain Receptor Tyrosine Kinases: New Players in Cancer Progression. Cancer Metastasis Rev..

[7] Vogel W.F., Aszódi A., Alves F., Pawson T. (2001). Discoidin Domain Receptor 1 Tyrosine Kinase Has an Essential Role in Mammary Gland Development. Mol. Cell. Biol..

[8] Vella V., Nicolosi M.L., Giuliano M., Morrione A., Malaguarnera R., Belfiore A. (2019). Insulin Receptor Isoform A Modulates Metabolic Reprogramming of Breast Cancer Cells in Response to IGF2 and Insulin Stimulation. Cells.

[9] Vella V., Malaguarnera R., Nicolosi M.L., Palladino C., Spoleti C., Massimino M., Vigneri P., Purrello M., Ragusa M., Morrione A. (2017). Discoidin Domain Receptor 1 Modulates Insulin Receptor Signaling and Biological Responses in Breast Cancer Cells. Oncotarget.

[10] Vella V., Nicolosi M.L., Cantafio P., Massimino M., Lappano R., Vigneri P., Ciuni R., Gangemi P., Morrione A., Malaguarnera R. (2019). DDR1 Regulates Thyroid Cancer Cell Differentiation via IGF-2/IR-A Autocrine Signaling Loop. Endocr. Relat. Cancer.

[11] Vella V., Malaguarnera R., Nicolosi M.L., Morrione A., Belfiore A. (2019). Insulin/IGF Signaling and Discoidin Domain Receptors: An Emerging Functional Connection. Biochim. Biophys. Acta Mol. Cell Res..

[12] Malaguarnera R., Nicolosi M.L., Sacco A., Morcavallo A., Vella V., Voci C., Spatuzza M., Xu S.-Q., Iozzo R.V., Vigneri R. (2015). Novel Cross Talk between IGF-IR and DDR1 Regulates IGF-IR Trafficking, Signaling and Biological Responses. Oncotarget.

[13] Belfiore A., Malaguarnera R., Nicolosi M.L., Lappano R., Ragusa M., Morrione A., Vella V. (2018). A Novel Functional Crosstalk between DDR1 and the IGF Axis and Its Relevance for Breast Cancer. Cell Adhes. Migr..

[14] Matà R., Palladino C., Nicolosi M.L., Lo Presti A.R., Malaguarnera R., Ragusa M., Sciortino D., Morrione A., Maggiolini M., Vella V. (2016). IGF-I Induces Upregulation of DDR1 Collagen Receptor in Breast Cancer Cells by Suppressing MIR-199a-5p through the PI3K/AKT Pathway. Oncotarget.

[15] Buraschi S., Morcavallo A., Neill T., Stefanello M., Palladino C., Xu S.-Q., Belfiore A., Iozzo R.V., Morrione A. (2020). Discoidin Domain Receptor 1 Functionally Interacts with the IGF-I System in Bladder Cancer. Matrix Biol. Plus.

[16] Gao H., Chakraborty G., Zhang Z., Akalay I., Gadiya M., Gao Y., Sinha S., Hu J., Jiang C., Akram M. (2016). Multi-Organ Site Metastatic Reactivation Mediated by Non-Canonical Discoidin Domain Receptor 1 Signaling. Cell.

[17] Gao Y., Zhou J., Li J. (2020). Discoidin Domain Receptors Orchestrate Cancer Progression: A Focus on Cancer Therapies. Cancer Sci..

[18] Xie X., He H., Zhang N., Wang X., Rui W., Xu D., Zhu Y. (2020). Overexpression of DDR1 Promotes Migration, Invasion, Though EMT-Related Molecule Expression and COL4A1/DDR1/MMP-2 Signaling Axis. Technol. Cancer Res. Treat..

[19] Sun L., Suo C., Li S.-T., Zhang H., Gao P. (2018). Metabolic Reprogramming for Cancer Cells and Their Microenvironment: Beyond the Warburg Effect. Biochim. Biophys. Acta Rev. Cancer.

[20] Warburg O., Wind F., Negelein E. (1927). The Metabolism of Tumors in the Body. J. Gen. Physiol..

[21] Bose S., Le A. (2018). Glucose Metabolism in Cancer. Adv. Exp. Med. Biol..

[22] Gatenby R.A., Gillies R.J. (2004). Why Do Cancers Have High Aerobic Glycolysis?. Nat. Rev. Cancer.

[23] Valcarcel-Jimenez L., Gaude E., Torrano V., Frezza C., Carracedo A. (2017). Mitochondrial Metabolism: Yin and Yang for Tumor Progression. Trends Endocrinol. Metab. TEM.

[24] L’hôte C.G.M., Thomas P.H., Ganesan T.S. (2002). Functional Analysis of Discoidin Domain Receptor 1: Effect of Adhesion on DDR1 Phosphorylation. FASEB J. Off. Publ. Fed. Am. Soc. Exp. Biol..

[25] Krzeslak A., Wojcik-Krowiranda K., Forma E., Jozwiak P., Romanowicz H., Bienkiewicz A., Brys M. (2012). Expression of GLUT1 and GLUT3 Glucose Transporters in Endometrial and Breast Cancers. Pathol. Oncol. Res. POR.

[26] Hamann I., Krys D., Glubrecht D., Bouvet V., Marshall A., Vos L., Mackey J.R., Wuest M., Wuest F. (2018). Expression and Function of Hexose Transporters GLUT1, GLUT2, and GLUT5 in Breast Cancer-Effects of Hypoxia. FASEB J. Off. Publ. Fed. Am. Soc. Exp. Biol..

[27] Halestrap A.P., Price N.T. (1999). The Proton-Linked Monocarboxylate Transporter (MCT) Family: Structure, Function and Regulation. Biochem. J..

[28] Rofstad E.K., Mathiesen B., Kindem K., Galappathi K. (2006). Acidic Extracellular PH Promotes Experimental Metastasis of Human Melanoma Cells in Athymic Nude Mice. Cancer Res..

[29] Malaguarnera R., Belfiore A. (2011). The Insulin Receptor: A New Target for Cancer Therapy. Front. Endocrinol..

[30] Gleyzer N., Vercauteren K., Scarpulla R.C. (2005). Control of Mitochondrial Transcription Specificity Factors (TFB1M and TFB2M) by Nuclear Respiratory Factors (NRF-1 and NRF-2) and PGC-1 Family Coactivators. Mol. Cell. Biol..

[31] Amoedo N.D., Punzi G., Obre E., Lacombe D., De Grassi A., Pierri C.L., Rossignol R. (2016). AGC1/2, the Mitochondrial Aspartate-Glutamate Carriers. Biochim. Biophys. Acta.

[32] Baldelli S., Aquilano K., Ciriolo M.R. (2013). Punctum on Two Different Transcription Factors Regulated by PGC-1α: Nuclear Factor Erythroid-Derived 2-like 2 and Nuclear Respiratory Factor 2. Biochim. Biophys. Acta.

[33] Luo C., Widlund H.R., Puigserver P. (2016). PGC-1 Coactivators: Shepherding the Mitochondrial Biogenesis of Tumors. Trends Cancer.

[34] Ma Y., Wang L., Jia R. (2020). The Role of Mitochondrial Dynamics in Human Cancers. Am. J. Cancer Res..

[35] Belfiore A., Malaguarnera R., Vella V., Lawrence M.C., Sciacca L., Frasca F., Morrione A., Vigneri R. (2017). Insulin Receptor Isoforms in Physiology and Disease: An Updated View. Endocr. Rev..

[36] Zhong X., Zhang W., Sun T. (2019). DDR1 Promotes Breast Tumor Growth by Suppressing Antitumor Immunity. Oncol. Rep..

[37] Ongusaha P.P., Kim J., Fang L., Wong T.W., Yancopoulos G.D., Aaronson S.A., Lee S.W. (2003). P53 Induction and Activation of DDR1 Kinase Counteract P53-Mediated Apoptosis and Influence P53 Regulation through a Positive Feedback Loop. EMBO J..

[38] Das S., Ongusaha P.P., Yang Y.S., Park J.-M., Aaronson S.A., Lee S.W. (2006). Discoidin Domain Receptor 1 Receptor Tyrosine Kinase Induces Cyclooxygenase-2 and Promotes Chemoresistance through Nuclear Factor-KappaB Pathway Activation. Cancer Res..

[39] Cader F.Z., Vockerodt M., Bose S., Nagy E., Brundler M.-A., Kearns P., Murray P.G. (2013). The EBV Oncogene LMP1 Protects Lymphoma Cells from Cell Death through the Collagen-Mediated Activation of DDR1. Blood.

[40] Belfiore A., Frasca F. (2008). IGF and Insulin Receptor Signaling in Breast Cancer. J. Mammary Gland Biol. Neoplasia.

[41] Milazzo G., Giorgino F., Damante G., Sung C., Stampfer M.R., Vigneri R., Goldfine I.D., Belfiore A. (1992). Insulin Receptor Expression and Function in Human Breast Cancer Cell Lines. Cancer Res..

[42] Sciacca L., Costantino A., Pandini G., Mineo R., Frasca F., Scalia P., Sbraccia P., Goldfine I.D., Vigneri R., Belfiore A. (1999). Insulin Receptor Activation by IGF-II in Breast Cancers: Evidence for a New Autocrine/Paracrine Mechanism. Oncogene.

[43] Papa V., Pezzino V., Costantino A., Belfiore A., Giuffrida D., Frittitta L., Vannelli G.B., Brand R., Goldfine I.D., Vigneri R. (1990). Elevated Insulin Receptor Content in Human Breast Cancer. J. Clin. Investig..

